# Carotid and regional arterial stiffness and dementia‐related imaging biomarkers in the Multi‐Ethnic Study of Atherosclerosis (MESA)

**DOI:** 10.1002/alz.70688

**Published:** 2025-10-17

**Authors:** Sheina Emrani, Jordan Tanley, Christopher L. Schaich, Sanjiv Shah, Alain G. Bertoni, Claudia Korcarz, Susan R. Heckbert, Mohamad Habes, Samuel N. Lockhart, Julio A. Chirinos, Jingzhong Ding, James H. Stein, Adam D. Gepner, R. Nick Bryan, Ilya M. Nasrallah, José A. Luchsinger, Kathleen M. Hayden, Yongmei Liu, Timothy M. Hughes

**Affiliations:** ^1^ Department of Neurology Perelman Center for Advanced Medicine University of Pennsylvania Philadelphia Pennsylvania USA; ^2^ Department of Internal Medicine, Section on Gerontology and Geriatric Medicine Wake Forest School of Medicine Winston‐Salem North Carolina USA; ^3^ Department of Surgery Hypertension and Vascular Research Center Wake Forest University School of Medicine Winston‐Salem North Carolina USA; ^4^ Department of Cardiology Northwestern University School of Medicine Chicago Illinois USA; ^5^ Division of Public Health Sciences Wake Forest University School of Medicine Winston‐Salem North Carolina USA; ^6^ Cardiovascular Division, Department of Medicine University of Wisconsin School of Medicine and Public Health Madison Wisconsin USA; ^7^ Department of Epidemiology University of Washington School of Public Health Seattle Washington USA; ^8^ Neuroimage Analytics Laboratory and Biggs Institute Neuroimaging Core Glenn Biggs Institute for Alzheimer's & Neurodegenerative Diseases University of Texas Health Science Center San Antonio Texas USA; ^9^ Division of Cardiovascular Medicine Hospital of the University of Pennsylvania Philadelphia Pennsylvania USA; ^10^ Department of Medicine Division of Cardiovascular Medicine William S. Middleton Memorial Veteran's Hospital Madison Wisconsin USA; ^11^ Department of Radiology University of Pennsylvania Philadelphia Pennsylvania USA; ^12^ Departments of Medicine and Epidemiology Columbia University Irving Medical Center New York New York USA; ^13^ Department of Social Sciences and Health Policy Division of Public Health Sciences Wake Forest School of Medicine Winston‐Salem North Carolina USA; ^14^ Department of Medicine Division of Cardiology Duke Molecular Physiology Institute Duke University Medical Center Durham North Carolina USA

**Keywords:** amyloid beta deposition, arterial stiffness, cerebrovascular disease, neuroimaging

## Abstract

**INTRODUCTION:**

Arterial stiffness measured within various arterial beds may be differentially associated with neuroimaging biomarkers of dementia.

**METHODS:**

We related carotid and regional (cardio‐ankle vascular index [CAVI] and heart‐ankle pulse wave velocity [haPWV]) arterial stiffness measures to biomarkers (gray matter volume [GMV], white matter hyperintensity volume [WMHV], and fractional anisotropy [WMFA]) and amyloid positron emission tomography (PET) positivity (centiloid > 12.2), controlling for covariates.

**RESULTS:**

All arterial stiffness measures were associated with higher WMHV. Lower carotid distensibility (increased mechanical stress) was positively associated with WMFA, while Young's elastic modulus and haPWV (greater stiffness) were associated with lower WMFA. Only CAVI was significantly related to amyloid PET positivity, although similar effect sizes were observed for carotid measures. No main associations were observed with GMV. Significant interactions showed men and Black and Hispanic participants had stronger associations between carotid stiffness and GMV.

**CONCLUSIONS:**

Carotid stiffness measures were associated with WM injury while regional CAVI measures were associated with amyloid positivity.

**Highlights:**

- We studied differences between carotid ultrasound and regional (cardio‐ankle vascular index [CAVI] and heart‐ankle pulse wave velocity [haPWV]) measures of arterial stiffness and neuroimaging abnormalities (white matter changes and amyloid positron emission tomography [PET] positivity) in the Multi‐Ethnic Study of Atherosclerosis, a diverse cohort of older adults.
- Carotid measures were associated with white matter injury, demonstrated usingwhite matter hyperintensity volume and white matter fractional anisotropy, and were not associated with amyloid PET positivity.
- Regional measures had variable relationships with white matter injury and CAVI, and in particular, were associated with amyloid deposition.
- Black and Hispanic participants had significant associations between arterial stiffness measures and brain volume that were not observed in White participants.
- Men showed significant associations with carotid distensibility and white matter injury while women did not.

## 1 INTRODUCTION

Cardiovascular disorders are important modifiable risk factors for Alzheimer's disease (AD) and AD‐related dementias (ADRD).^1^, ^2^, ^3^ Of all modifiable vascular risk factors, midlife hypertension appears to have one of the strongest effects on dementia risk,^4^ presumably through hemodynamic effects on the brain and its vasculature. In a randomized study of hypertension treatment, the Systolic Blood Pressure Intervention Trial (SPRINT) showed that relative to standard blood pressure targets, an intensive lowering of blood pressure resulted in significant reductions in cardiovascular events, reduced risk of cognitive impairment, and slowed rates of white matter disease development, typically observed as downstream effects of cardiovascular disease.^5^, ^6^ Nonetheless, while there has been progress in developing interventions to reduce blood pressure, the mechanism by which blood pressure may contribute to dementia and AD‐related pathologies remains unclear.

Emerging evidence shows that arterial stiffness precedes the development of hypertension, diabetes, kidney disease, and dementia,^7^, ^8^ likely through increases in large artery regional stiffness, followed by other vascular beds, leading to abnormalities in distal perfusion and end‐organ damage.^9^ Arterial stiffness is commonly measured by carotid ultrasound (e.g., distensibility and stiffness modulus) and tonometry‐based methods (e.g., pulse wave velocity [PWV]). The former method is based on quantifying the distension associated with variations in pressure, whereas the latter is based on quantification of the speed of wave transit along the arterial wall. Large artery stiffness measured by PWV has been associated with cognitive decline, incident cognitive impairment, abnormalities in cerebral perfusion and other forms of dementia‐related pathology, including multiple forms of cerebral small vessel disease and beta‐amyloid (Aβ) deposition and accumulation over time, elevated tau deposition,^10^ and neuroinflammation^11^ in older adults free from dementia.^9^, ^12^, ^13^, ^14^, ^15^, ^16^ Measures of arterial stiffness in other vascular beds, including carotid distensibility, a measure of carotid artery elasticity and stiffness, or wall rigidity, may also be associated with dementia pathology.^11^ Yet, few studies have evaluated arterial stiffness across multiple vascular beds to determine the potential for differential relationships to AD/ADRD neuroimaging biomarkers.

**RESEARCH IN CONTEXT**

1. **Systematic review**: We performed a literature search using traditional sources (e.g., PubMed, Google Scholar). Recent studies have shown both regional and carotid measures of arterial stiffness to be associated with neuroimaging abnormalities in Alzheimer's disease and related dementias (ADRD). Few studies have compared these arterial stiffness measures in relation to ADRD‐related brain changes.
2. **Interpretation**: Using the Multi‐Ethnic Study of Atherosclerosis (MESA), a diverse cohort of older adults, we show that all arterial stiffness measures were associated with white matter injury, although carotid measures were more sensitive. Comparatively, a regional measure of arterial stiffness was associated with amyloid positron emission tomography (PET) positivity, while carotid measures were not. No associations were observed with brain volumes. Finally, significant interactions showed men and Black and Hispanic participants had stronger associations between arterial stiffness and brain volume. These findings are important for understanding biological contributions to neuroimaging abnormalities in ADRD and leveraging sensitive tools to detect ADRD‐related brain changes, both crucial for prevention and treatment strategies.
3. **Future directions**: Future research will examine regional stiffness associations of brain morphology and cerebral blood flow, as well as other measures of cerebral small vessel disease to more precisely capture vulnerable areas. Additionally, future studies will expand upon racially/ethnically diverse individuals to inclusion of other groups in the MESA cohort, including Chinese Americans.

Although limited in scope, carotid arterial stiffness may be more sensitive to cerebral hemodynamics and cerebral small vessel disease pathology compared to aorta‐centric PWV measures of arterial stiffness.^17^ Additionally, less data are available to determine whether the observed associations between arterial stiffness and brain health are consistent across racial/ethnic groups, gender, and in those with an inherited risk of dementia by way of the apolipoprotein epsilon E (*APOE*‐ε4) or a family history of dementia. Therefore, more precise pathology‐dependent measures are needed to relate arterial stiffness to cerebrovascular function that can also extrapolate these relationships when considering genetic and social factors, stratified by genetic, gender, and racial/ethnic differences.

Given the lack of direct comparisons of carotid and regional stiffness measures to neuroimaging aspects of AD/ADRD in the same participants and influence of demographic and genetic factors on arterial stiffness, our study seeks to investigate these differences and relationships. Findings could have meaningful clinical implications in identifying populations for whom regional versus carotid stiffness measures may be most informative in relation to AD/ADRD‐related changes. Therefore, the goals of this study were to examine arterial stiffness measures across various arterial beds, including carotid ultrasound and regional cardio‐ankle vascular index (CAVI), in relation to brain atrophy and small vessel disease changes measured by magnetic resonance imaging (MRI) and Aß deposition from amyloid PET, in a diverse multisite cohort of older adults. Our overall hypothesis was that both carotid (Young's elastic modulus [YEM]) and regional (CAVI and cardio‐ankle pulse‐wave velocity) measures of arterial stiffness associate with greater atrophy, burden of cerebral small vessel disease, and amyloid deposition, while the distensibility coefficient would associate in the opposite direction. Because of the closer proximity to the brain, we hypothesize that carotid stiffness measures would be more strongly associated with these brain imaging measures compared to regional stiffness measures.

## 2 METHODS

The data used in this analysis are available through the Multi‐Ethnic Study of Atherosclerosis (MESA) Coordinating Center with an approved paper proposal. Instructions for data access may be found at https://www.mesa‐nhlbi.org/.

### 2.1 Study population

MESA is a prospective study of subclinical atherosclerosis among 6814 women and men. Forty‐five to eighty‐four years of age recruited from six centers: Baltimore, Maryland; Chicago, Illinois; Forsyth County, North Carolina; Los Angeles County, California; New York, New York; and St. Paul, Minnesota. Participants were free of clinical diagnoses of cardiovascular disease at baseline. Participants self‐identified as Black, Chinese, Hispanic, or White, and sites balanced recruitment between two or more racial and ethnic groups. Study objectives and details have been previously reported.^18^ The baseline exam for MESA occurred between 2000 and 2002, and six follow‐up MESA exams have been performed, including Exam 6 (2016–2018). Brain imaging was completed with magnetic resonance imaging (MRI; 2017–2021) and positron emission tomography (PET; 2017–2021).

### 2.2 B‐mode carotid ultrasound and brachial blood pressure measurements

**FIGURE 1:**
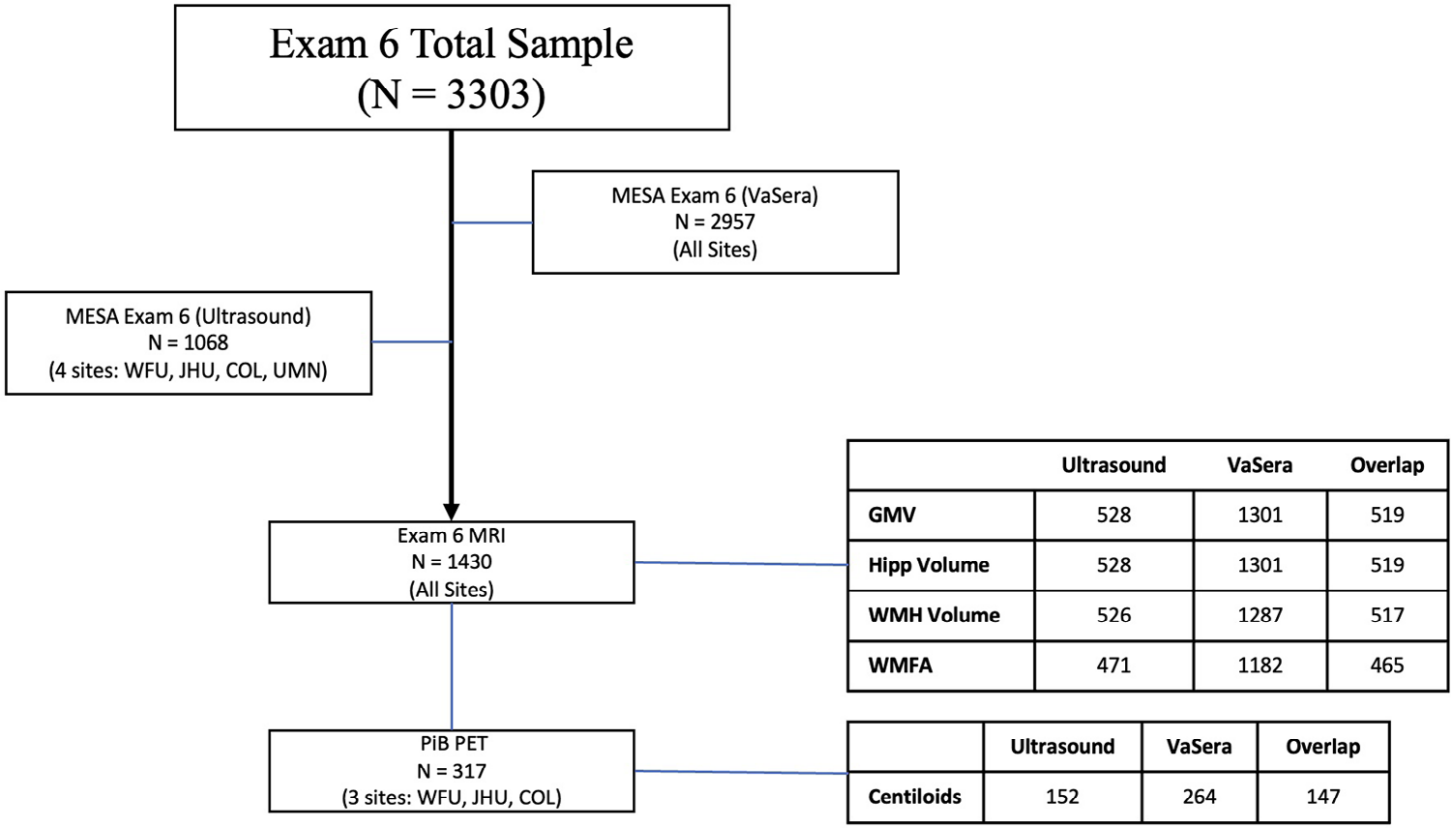
Flowchart. WFU, Wake Forest University; JHU, John Hopkins University; COL, Columbia University; UMN, University of Minnesota; Hipp, hippocampal; GMV, gray matter volume; WMH, white matter hyperintensity; WMFA, white matter fractional anisotropy.

At Exam 6, participants underwent carotid ultrasound and VaSera studies at four sites (see Figure 1 for flow chart of design and sample sizes) to derive arterial stiffness measures. Not all MESA sites participated in carotid ultrasound at Exam 6, especially those enrolling Chinese American participants, who were ultimately excluded from all analyses that examined race/ethnicity. Digital B‐mode ultrasound four‐beat loop recordings of a longitudinal section of the distal right common carotid artery were obtained using Mindray M9 ultrasound machines and the L12‐4s (Mindray, Mahwah, NJ). Sonographers from the four participating MESA sites who process these measurements were centrally trained and completed scanning certification. Following 10 min of rest in the supine position and immediately before ultrasound image acquisition, repeated measures of brachial blood pressures were gathered using a standardized protocol with an automated upper arm sphygmomanometer (DINAMAP; GE Medical Systems, Milwaukee, WI). Ultrasound images were reviewed and interpreted by the MESA Carotid Ultrasound Reading Center (the University of Wisconsin Atherosclerosis Imaging Research Program Lab, Madison, WI). Systolic and diastolic diameters were set as the largest and smallest diameters during the cardiac cycle. All measurements were made by manually tracing a 1 cm long segment and performed in triplicate from two to three consecutive cardiac cycles. Carotid artery diameters and thicknesses were measured using Access Point Web version 8.2 (Freeland Systems LLC, Carmel, IN).

Carotid artery distensibility coefficient (DC) and YEM were calculated using standard accepted formula^19^, ^20^ and have been reported previously.^21^

$$\mathrm{DC}:\frac{\left(Ds^{2}-Dd^{2}\right)}{\Delta p\;\cdot \;Dd^{2}}$$

*Ds* represents the internal arterial diameter at peak systole, *Dd* represents the internal diameter at end‐diastole, and Δ*p* represents the difference between the systolic and diastolic measurements (pulse pressure).

YEM was calculated using the following formula:

$$\mathrm{YEM}\;=\frac{Dd/h}{\mathrm{DC}}$$

*Dd* is the arterial diameter at end‐diastole and *h* is the arterial wall thickness at end‐diastole (wall thickness was calculated as external diameter minus internal diameter of the distal common carotid artery).^21^ YEM and DC are inversely related; thus, increased arterial stiffness corresponds to a lower DC and a higher YEM.

### 2.3 CAVI

At Exam 6, participants underwent arterial stiffness assessments at all sites using the VaSera 1500N device (Fukuda Denshi, Tokyo, Japan). The VaSera system estimates heart‐ankle pulse wave velocity (haPWV) by combining the heart‐to‐brachial transit time, calculated as the time difference between the closing of the aortic valve (from the phonocardiogram) and the dicrotic notch on the brachial cuff signal, as well as the brachial‐to‐ankle transit time, calculated as the foot‐to‐foot time difference between the brachial and ankle cuffs. The VaSera Data Management Software VSS‐10U (Fukuda Denshi) was used to analyze data. Similar to the carotid ultrasound protocol, participants rested for 10 min in the supine position. Measurements were taken using two brachial and two ankle cuffs (one per side), and a phonocardiograph, based on manufacturer's instructions. Cuffs were placed approximately 2 cm above the medial malleolus on the legs and about 2 cm above the antecubital fossa on the arms. Cuff size was adjusted according to arm size, per the manufacturer's instructions. VaSera measures, or regional stiffness measures, reported in this study include haPWV and CAVI, where higher levels are indicative of greater arterial stiffness along the heart‐ankle path. Information regarding CAVI and PWV calculations can be found elsewhere.^22^, ^23^ Briefly, heart‐ankle PWV was calculated as the distance between the heart and ankle divided by differences in conduction time. The mathematical expression to calculate CAVI is based on the following equation: *β* = 2*ρ*×1/(SBP–DBP)×ln (SBP/DBP)×PWV^2^, where *ρ* is the blood density, PWV is the heart‐ankle PWV, SBP is systolic blood pressure, and DBP is diastolic blood pressure.^24^

### 2.4 Risk factors

Self‐reported age, gender, race/ethnicity, and education level were collected at baseline. The self‐reported race and ethnicity categories were Black, Chinese, Hispanic, and White. Information is not available on the self‐reported race of participants who identified as Hispanic. All other risk factor data were collected at Exam 6 (2016–2018). Dynamic factors, like smoking status, and medication use, were updated, as necessary. Telephone contacts were made every 9–12 months during follow‐up after the baseline visit by study staff to identify new hospitalizations and diagnoses. Height and weight were measured by study staff. Blood pressure was calculated as the average of the last two of three seated measurements. Hypertension was defined as SBP ≥ 140 mm Hg, DBP ≥ 90 mm Hg, or use of antihypertensive medications as defined by the 2003 Joint National Committee guidelines.^25^ Diabetes was defined as use of diabetes medication, fasting glucose ≥ 126 mg/dL, or hemoglobin A1c ≥ 6.5. Medical records were obtained and new onset myocardial infarction, heart failure, atrial fibrillation and stroke between baseline and Exam 6 were ascertained, as previously reported.^18^, ^26^, ^27^ Participants were asked to self‐report a family history of AD or senile dementia in a first‐degree relative (e.g., mother, father, biological sibling). *APOE* isoforms were estimated from single nucleotide polymorphisms rs429358 and rs7412 from the genotyping conducted in all MESA participants.

### 2.5 Brain MRI acquisition and processing

Brain MRI scans were acquired on 3‐Tesla (3T) Siemens scanners: Prisma VE11C (University of California Los Angeles, Columbia University, John Hopkins University, Northwestern University, University of Minnesota) and Skyra VD11B (University of California Los Angeles, Wake Forest University). Initial MRIs were collected on 1430 MESA participants between 2017 and 2022. Staff from the University of Pennsylvania Brain MRI Reading Center supervised study quality and trained MRI technologists to perform standardized imaging protocols. Structural MRI brain sequences included 1 mm isotropic sagittal 3D T1‐weighted, T2‐weighted, and axial 2D echo‐planar diffusion‐tensor imaging (DTI). A detailed brain MRI protocol can be found elsewhere.^28^

### 2.6 MRI measures

Variables of interest derived from the MRI images included total gray matter volume (GMV), total hippocampal volume, total white matter hyperintensity volume (WMHV), and total WM fractional anisotropy (FA). Automated pipelines were applied for preprocessing structural MRIs, including inhomogeneity correction^29^ and multi‐atlas skull‐stripping,^30^ and region of interest quantification using MUlti‐atlas region Segmentation utilizing Ensembles (MUSE).^31^ Total intracranial volume was defined as the sum of all GM, WM, and cerebrospinal fluid. Using a deep learning‐based segmentation method,^32^ total WMHV was measured from inhomogeneity corrected and co‐registered fluid attenuated inversion recovery and T1‐weighted images. Finally, WMFA is a measure of WM integrity calculated from DTI using automated pipelines.^33^ FA is the degree to which water diffusion is limited to a single dimension. This variable is scalar ranging from 0, indicating equivalent motion in all directions, to 1, indicating motion restricted to a single direction. Lowered FA are interpreted as demonstrating disrupted WM integrity and indicative of WM injury that can be associated with cerebral small vessel disease.

### 2.7 Amyloid PET imaging

Amyloid PET imaging was performed with [11C] Pittsburgh Compound B (PiB) at the Wake Forest site at Exam 6. [11C]PiB acquisition methods have been described previously.^34^, ^35^, ^36^ Following a computed tomography (CT) scan for attenuation correction, participants were injected with ∼10mCi [11C]PiB and scanned 40–70 min (6 × 5‐min frames) post‐injection on a 64‐slice GE Discovery MI DR PET/CT scanner. In MESA MIND Visit A (2019‐2021), amyloid PET imaging was extended to Columbia and Johns Hopkins sites using Siemens Biograph mCT PET/CT scanners using [18F] Florbetaben (FBB) or [11C]PiB ([11C]PiB imaging parameters were the same as Wake Forest; Johns Hopkins University also used [18F]FBB: ∼8 mCi dose with scanning at 90–110 min (4 × 5‐min frames) post‐injection). Each participant's CT image was co‐registered to their structural MRI, and PET frames were co‐registered to MRI space using the affine matrix from the CT‐MRI co‐registration. Centiloid (CL) values, which are a tracer‐agnostic, scaled measure of global cerebral amyloid burden, were calculated using standard methods to allow harmonization across ligands.^37^ Amyloid PET studies were considered positive when CL measures were above 12.2.^38^

## 3 STATISTICAL ANALYSES

Descriptive statistics were examined within each analytic sample. All arterial stiffness measures were standardized for ease of comparisons. For our primary objective to assess the relationship between carotid ultrasound measures and brain imaging outcomes, we excluded 28 participants whose MRI images did not meet quality control criteria (i.e., poor quality or missing imaging, or presence of structural lesions). Additionally, two participants who identified as Chinese were excluded when employing analyses that included race, given the lack of carotid ultrasound measures in this group. To investigate the relationship between arterial stiffness measures and brain imaging outcomes, we used multivariable generalized linear models with each stiffness measure as the predictor and each MRI outcome as the outcome variable, adjusting for two levels of model. WMHV was log transformed due to skewness. Logistic regression was employed for amyloid PET analyses, with the same modeling described below. The basic model (Model 1) included age at Exam 6, gender, race/ethnicity, *APOE*e4 status (presence/absence of *APOE*‐ε4 allele), study site, and, for MRI‐based volumetric variables, total intracranial volume. A second model (Model 2) included additional adjustment for smoking history, education, systolic blood pressure, diabetes status, and use of anti‐hypertensive medication.^34^, ^39^, ^40^ Secondary analysis examined the moderation of each stiffness measure and brain imaging measures by the covariates described above, with the exception of Site. In these analyses, age was dichotomized by median split (below and above the age of 73) to capture high versus low age risk. For statistically significant findings, additional linear model analysis was completed, stratified by the covariate. We used SASv9.4 for analysis.

## 4 RESULTS

At Exam 6 of MESA, 2982 participants completed a vascular assessment: 2957 individuals had VaSera (regional) measures, 1068 had carotid ultrasound measures, and 1041 had both regional arterial stiffness and carotid ultrasound (see Table S1 for breakdown of the subset individuals with both arterial stiffness measures and neuroimaging). Average age at Exam 6 of the entire sample was 73 years and most participants had at least a high school degree. The overall sample was 53% women and 40% were White, 13% Chinese, 23% Black, and 22% Hispanic individuals. The demographics of those with neuroimaging data (Regional; *n* = 1301 for total GMV, *n* = 1287 for WMHV, *n* = 264 for PET; Carotid; *n* = 528 for total GMV, *n* = 526 for WMHV, *n* = 152 for PET) was similar among those with regional and carotid stiffness measures (see Table 1). Other information including family history of dementia, *APOE*‐ε4 genotype, arterial stiffness data, and neuroimaging as well as a breakdown of these variables across analytic sample can be found in Table 1. Figure S1 shows Pearson correlations between the arterial stiffness measures, with log transformed carotid ultrasound data. Overall, regional stiffness measures (CAVI and haPWV) showed a high positive correlation. Similarly, carotid stiffness measures (carotid distensibility and carotid YEM) showed a high negative correlation with each other. There were weak to moderate associations between carotid and regional stiffness measures. Associations of arterial stiffness measures across demographic factors from multivariable models can be found in Table S2. Our reference groups for analyses were based on who had the largest sample size or the absence of disease for comparisons. Less distensibility and greater measures of carotid and regional arterial stiffness were consistently associated with older age, men, Hispanic ethnicity, diabetes, higher systolic blood pressure, and use of antihypertensive medications.

**TABLE 1:** MESA Exam 6 demographic, vascular, and neuroimaging measures among participants who have neuroimaging data and arterial stiffness measures from VaSera (Regional; *N* = 1301) or carotid ultrasound (Carotid; *N* = 528).

		MESA participants with regional stiffness and neuroimaging data			MESA participants with carotid stiffness and neuroimaging data		
		(*n* = 1301)			(*n* = 528)		
Parameter		Total N	N / Mean / Median	% / SD / IQR	Total N	N / Mean / Median	% / SD / IQR
Age Exam 6	mean / SD	1301	72	8	528	72	8
Education							
≤ High school	n / %	1301	330	25%	528	134	25%
> High school			971	75%		394	75%
Gender							
Women	n / %	1301	690	53%	528	288	55%
Men			611	47%		240	45%
Race/ethnicity							
White	n / %	1301	547	42%	528	229	43%
Chinese			161	12%		0	0%
Black			342	26%		167	32%
Hispanic			251	19%		132	25%
Family history of dementia							
No	n / %	1301	961	74%	528	395	75%
Yes			340	26%		133	25%
*APOE*‐e4, ≥1 allele							
No	n / %	1301	903	69%	528	366	69%
Yes			339	26%		140	27%
Missing			59	5%		22	4%
Current smoking status							
No	n / %	1301	1228	94%	528	492	93%
Yes			73	6%		36	7%
Hypertension Medication							
No	n / %	1301	561	43%	528	227	43%
Yes			740	57%		301	57%
SBP (mmHg)	mean /SD	1301	126	20	528	128	19
Diabetes status							
Normal	n / %	1301	726	56%	528	302	57%
Impaired fasting glucose			304	23%		113	21%
Untreated diabetes			53	4%		21	4%
Treated diabetes			218	17%		92	17%
*Arterial Stiffness measures*							
Carotid distensibility (1/mmHg)	median / IQR	521	0.003	0.002	528	0.003	0.002
Carotid YEM (mmHg/mm)	median / IQR	521	1170	841	528	1176	849
haPWV (m/s)	median / IQR	1301	8.40	1.45	519	8.47	1.51
CAVI (units)	median / IQR	1301	8.94	1.66	519	9.05	1.65
*MRI*							
Total GMV (µl)	mean / SD	1301	596993	65924	528	595498	67389
Hippocampal volume (µl)	mean / SD	1301	3552	428	528	3523	427
WMHV (µl)	median / IQR	1287	2805	6342	526	2930	6176
WMFA	median / IQR	1182	0.39	0.04	471	0.40	0.04
*PET imaging*							
Amyloid PET Centiloids > 12.2							
No	n / %	264	184	70%	152	107	70%
Yes			80	30%		45	30%

*Note*: Hippocampal volume indicates mean bilateral hippocampal volume.

Abbreviations: APOE‐e4, apolipoprotein epsilon 4; CAVI, cardio‐ankle vascular index; GMV, gray matter volume; haPWV, heart‐ankle pulse wave velocity; IQR, interquartile range; MESA, Multi‐Ethnic Study of Atherosclerosis; MRI, magnetic resonance imaging; PET, positron emission tomography; SBP, systolic blood pressure; SD, standard deviation; WMFA, white matter fractional anisotropy; WMHV, white matter hyperintensity volume; YEM, Young's elastic modulus.

Greater carotid distensibility, indicating lower carotid stiffness, was associated with better white matter structural integrity indicated by lower WMHV and greater WMFA (Table 2). In basic and fully adjusted models, higher carotid distensibility was associated with less WMHV (fully adjusted model; Beta = −0.14 (SE = 0.07); *p* = 0.031) and, in the basic model only, greater WMFA (basic model; Beta = 0.003(SE = 0.001); *p* = 0.033). Similarly, greater carotid YEM, or lower carotid stiffness, was associated with lower WMHV (fully adjusted model; Beta = 0.16 (SE = 0.06); *p* = 0.012) and greater WMFA (fully adjusted model; Beta = ‐0.003 (SE = 0.001); *p* = 0.033). For measures of regional arterial stiffness derived from the VaSera device, decreased heart‐ankle PWV (decreased stiffness) was associated with lower WMHV in basic and fully adjusted models (Beta = 0.10 (SE = 0.04); *p* = 0.015) and greater WMFA in basic model only (Beta = ‐0.003 (SE = 0.001); *p* < 0.001). Lower CAVI (lower stiffness) was associated with lower WMHV in the basic model only (Beta = 0.082 (SE = 0.04); *p* = 0.03) (Table 2). No statistically significant findings were observed for the volumetric measures of total GMV or mean bilateral hippocampal volume within the total analytic sample (Table 2).

**TABLE 2:** Associations between arterial stiffness measures and MRI outcomes.

	Total GMV (mm^3^)			Hippocampal volume (mm^3^)			WMHV^a^			WMFA		
	Estimate	SE	*p*‐value	Estimate	SE	*p*‐value	Estimate	SE	*p*‐value	Estimate	SE	*p*‐value
Arterial stiffness measure	Carotid measures (*N* = 528)						Carotid measures (*N* = 526)			Carotid measures (*N* = 471)		
Carotid distensibility												
Model 1	1836	1465	0.211	−20.12	15.71	0.201	−0.18	0.06	**0.005**	0.003	0.001	**0.033**
Model 2	2368	1537	0.124	−7.62	16.56	0.646	−0.14	0.07	**0.031**	0.002	0.001	0.119
Carotid YEM												
Model 1	−1717	1434	0.232	15.84	15.38	0.304	0.19	0.06	**0.002**	−0.003	0.001	**0.006**
Model 2	−2297	1500	0.126	3.38	16.16	0.834	0.16	0.06	**0.012**	−0.003	0.001	**0.033**

Arterial stiffness measure	Regional measures (*N* = 1301)						Regional measures (*N* = 1287)			Regional measures (*N* = 1182)		
haPWV												
Model 1	−570	881	0.518	13.36	9.69	0.168	0.167	0.04	**<0.001**	−0.003	0.001	**<0.001**
Model 2	22	972	0.982	9.47	10.77	0.379	0.10	0.04	**0.015**	−0.001	0.001	0.104
CAVI												
Model 1	−140	880	0.873	12.46	9.67	0.198	0.082	0.04	**0.034**	−0.001	0.001	0.256
Model 2	323	885	0.715	11.80	9.80	0.229	0.053	0.04	0.177	−0.0002	0.001	0.823

*Note*: Model 1: Age at exam 6, Race/Ethnicity, Gender, APOE4, Site, ICV (For volumes). Model 2: Age at exam 6, Race/Ethnicity, Gender, APOE4, Site, Smoking status, education, systolic blood pressure, anti‐hypertension medication, diabetes status, ICV (for volumes). Carotid ultrasound full dataset; *N* = 1068; VaSera measures full dataset, *N* = 2955; Hippocampal volume indicates mean bilateral hippocampal volume.

The bolded numbers correspond to significant findings.

Abbreviations: CAVI, cardio‐ankle vascular index; GMV, gray matter volume; haPWV, heart‐ankle pulse wave velocity; ICV, intracranial volume; MRI, magnetic resonance imaging; SE, standard error; WMFA, white matter fractional anisotropy; WMHV, white matter hyperintensity volume; YEM, Young's elastic modulus.

^a^ ln(WMHV+0.001).

We next investigated the relationship of carotid and regional arterial stiffness measures had with cerebral amyloid deposition in a subset of our cohort (*n* = 152 [Carotid] and 264 [Regional]) with amyloid PET data. Regional stiffness measured by CAVI was associated with a higher odds of amyloid PET positivity (Beta = 1.40, 95% confidence interval [CI] (1.01–1.94); *p* = 0.048) (Table 3). No other significant association between other measures of stiffness and amyloid PET positivity were observed, although effect sizes of Carotid YEMS were similar to CAVI.

**TABLE 3:** Associations between arterial stiffness measures and amyloid PET positivity.

Amyloid PET Centiloids (>12.2)				
Parameter	Positive/ total	Odds ratio	95% CI	*p*‐value
**Carotid measures** (*N*=152)				
Carotid distensibility				
Model 1	45 / 152	0.77	(0.48, 1.22)	0.264
Model 2	45 / 152	0.74	(0.45, 1.21)	0.231
Carotid YEM				
Model 1	45 / 152	1.32	(0.82, 2.11)	0.251
Model 2	45 / 152	1.41	(0.86, 2.33)	0.176
**Regional measures** (*N*=264)				
haPWV				
Model 1	80 / 264	1.25	(0.89, 1.77)	0.199
Model 2	80 / 264	1.38	(0.94, 2.04)	0.099
CAVI				
Model 1	80 / 264	1.40	(1.01, 1.94)	**0.048**
Model 2	80 / 264	1.40	(0.99, 1.96)	**0.052**

*Note*: Model 1: Age at exam 6, Race/Ethnicity, Gender, Site, APOE4. Model 2: Age at Exam 6, race/ethnicity, gender, site, APOE4, smoking status, education, systolic blood pressure, hypertension medication, diabetes status.

The bolded numbers correspond to significant findings.

Abbreviations: CAVI, cardio‐ankle vascular index; CI, confidence interval; haPWV, heart‐ankle pulse wave velocity; PET, positron emission tomography; YEM, Young's elastic modulus.

Given the differences in sample sizes for carotid and regional stiffness measures, we conducted sensitivity analyses restricted to the subset of participants who had both carotid and regional stiffness measures. We found similar relationships observed in the full sample sizes for carotid and regional stiffness measures with MRI and PET (see Tables S3 and 4). For example, as seen in Table S3 for sensitivity analyses of carotid stiffness measures in relation to MRI outcomes in participants who had data available for all stiffness measures, only WMHV and WMFA were significantly associated to carotid stiffness measures. CAVI was no longer significantly associated with MRI measures. However, CAVI was significantly associated with amyloid PET imaging positivity in the basic and attenuated in fully adjusted models.

**FIGURE 2 alz70688-fig-0002:**
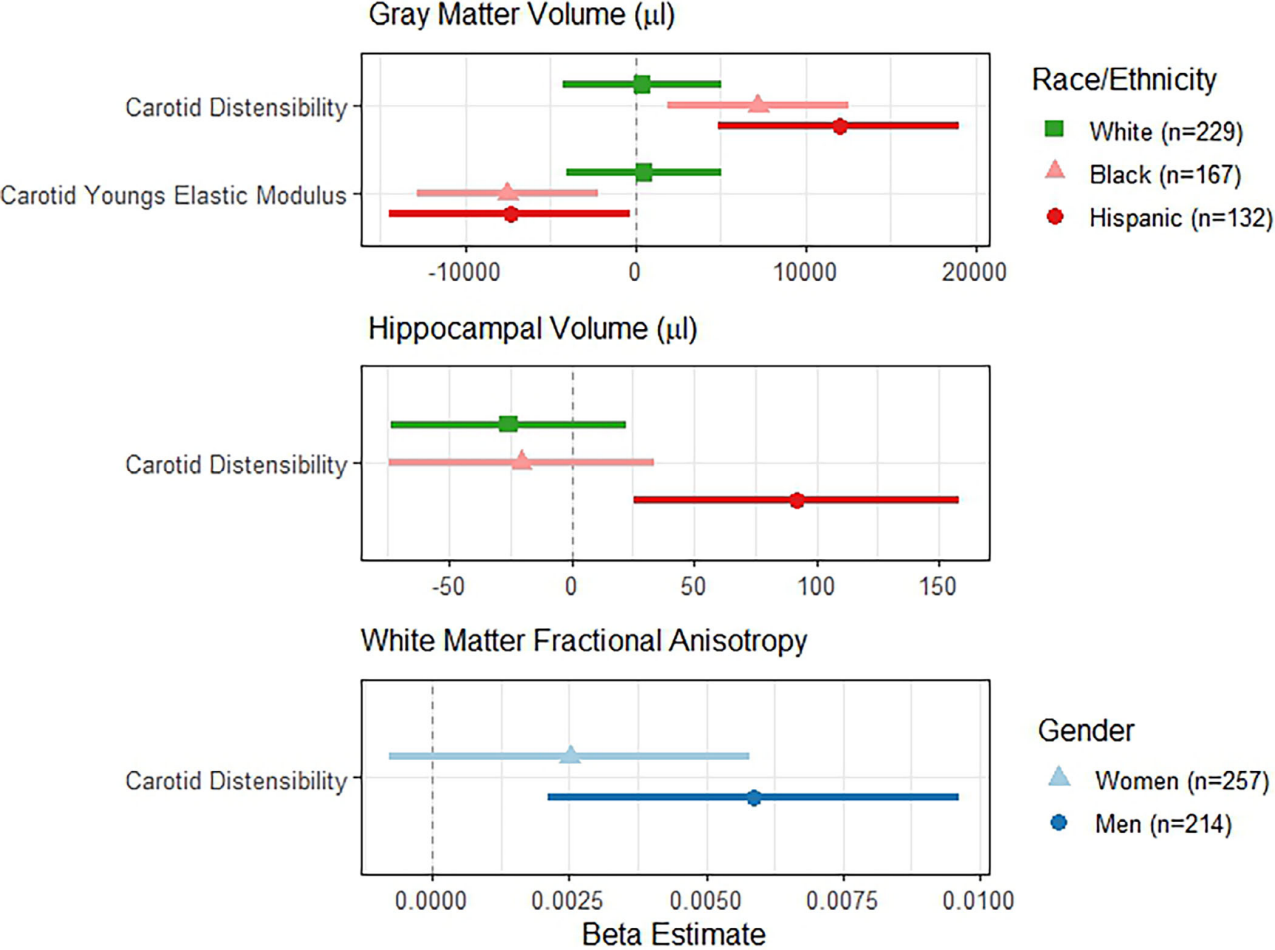
Forest plots for significant interactions with carotid ultrasound in models of MRI measures. MRI, magnetic resonance imaging.

**FIGURE 3 alz70688-fig-0003:**
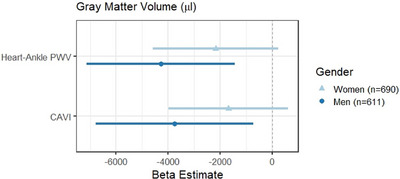
Forest plots for significant interactions with arterial stiffness measures in models of MRI Measures. CAVI, cardio‐ankle vascular index; MRI, magnetic resonance imaging; PWV, pulse wave velocity.

In the full sample, we investigated potential moderation by known risk factors for AD/ADRD, including age, *APOE*‐ε4 status, family history of dementia, gender, and race/ethnicity on the relationship between arterial stiffness and brain imaging using generalized linear models in individuals with carotid (Figure 2; Table S5) or regional stiffness data (Figure 3; Table S6). Figures 2 and 3 display point estimates and confidence intervals for the subgroups with observed significant interactions. The interaction between carotid stiffness measures and race/ethnicity was statistically significant for both carotid stiffness in relation to GMV and carotid distensibility only in relation to hippocampal volume. Specifically, Black and Hispanic individuals showed significant associations between greater carotid arterial stiffness and lower GMV that were not observed in White participants. Further, lower carotid distensibility was associated with smaller bilateral hippocampal volumes (Figure 2) in Hispanic participants. Meanwhile, the interaction between carotid distensibility and gender in relation to WMFA was statistically significant such that only men had a positive relationship between WMFA and carotid distensibility (lower stiffness associated with better white matter integrity). There was a significant interaction between regional stiffness and gender with GMV, as well (Figure 3; Table S6), such that associations between higher regional stiffness measures (both haPWV and CAVI) and lower GMV were driven by men (Figure 3).[Fig alz70688-fig-0002], [Fig alz70688-fig-0003]

## 5 DISCUSSION

In this diverse cohort of older adults, we examined cross‐sectional relationships between carotid (ultrasound) and regional (haPWV and CAVI; regional arterial segment) arterial stiffness measures with neuroimaging biomarkers of brain atrophy (GMV, hippocampal volume), white matter injury (WMHV and WMFA), and cerebral amyloid deposition. We found that carotid and regional arterial stiffness measures were associated with WMHV, with both carotid stiffness and haPWV showing a relationship with WMFA, though some analyses were attenuated in fully adjusted models that included cardiovascular diseases. CAVI, a measure of regional arterial stiffness, was strongly related to amyloid PET positivity while carotid stiffness was not. No associations were observed between arterial stiffness and GMV or hippocampal volume in the full sample. We observed significant relationships between carotid stiffness and GMV in Black and Hispanic participants and carotid distensibility and hippocampal volume in Hispanic participants that were not observed in White participants. Men showed significant associations between carotid distensibility and WM injury while women did not. These data suggest arterial stiffness may be important markers related to brain atrophy in men and Black and Hispanic older adults. More work is needed, as Black and Hispanic older adults are under‐represented in AD/ADRD research studies.

Overall, regional stiffness measures were more strongly associated with amyloid PET positivity than carotid stiffness measures, but exhibited weaker associations with WM integrity, especially in the presence of cardiovascular diseases. These findings confirm and extend prior studies showing that regional measures of arterial stiffness are more strongly associated with amyloid deposition^41^, ^42^ than carotid stiffness measures alone. Prior studies have shown carotid stiffness to be more strongly correlated with progression of cerebral Aβ deposition, while greater regional stiffness (composed of central elastic and peripheral muscular arteries) better reflects cerebral amyloid cross‐sectionally.^16^, ^43^

These findings underscore the value of looking at stiffness in different vascular beds. Age‐related arterial stiffness is proposed to begin in the aorta and progress to include the carotid and distal arterial beds before reaching the brain. Carotid ultrasound and tonometry‐based measure complementary aspects of arterial stiffness. Arterial distensibility is generally a measure of the arterial ability to expand and contract with cyclic variations in intraluminal pressure, with a decrease being indicative of arterial stiffness.^44^ Carotid YEM has been purported to measure subtle elastic properties of the carotid by taking arterial wall thickness into account. YEM measures stress‐strain relationship, or the ease of which arterial walls can bend or stretch. In contrast, regional measures of arterial stiffness assess stiffness by pulse wave velocity, usually across wide sections of vascular beds, such as the path originating from the aortic root to distal locations such as the tibial artery of the ankle. CAVI also has advantages of greater independence from blood pressure when using participant specific, corrected models for the effect of blood pressure on arterial stiffness, inclusion of ascending aorta in the area of measurement enabling more sensitive measures to cardiac function, and low cost and scalability.^45^, ^46^, ^47^, ^48^, ^49^ Other considerations when interpreting arterial stiffness measures include the effect of distending pressure (i.e., blood pressure) on wall material properties, including its PWV. When present in large regions of the body (e.g., CAVI), it may better represent systemic disease processes that produce aberrant hemodynamics as well as active biologic processes related to aging of the aortic wall^9^ and cerebral amyloid can accumulate over time through abnormalities in cerebral blood flow or disruptions in clearance mechanisms.^12^, ^14^, ^50^ These methods with detailed cognitive assessments of AD/ADRD biomarkers have recently been evaluated^51^ and should be considered in future studies. One possible explanation for the more consistent associations between regional arterial stiffness measures and amyloid deposition relative to carotid measures is that regional stiffness measures assess pulse propagation velocities, which may reflect pulsatile hemodynamics of end organs.

Black and Hispanic individuals showed associations between arterial stiffness and GMV not seen in White participants, similar to prior studies in Black^52^ and Hispanic^53^, ^54^ individuals. Additionally, Hispanic individuals showed greater associations between larger bilateral hippocampal volumes with increased carotid distensibility. Men had greater stiffness, lower distensibility, and were more likely to show associations with WM injury (lower FA) and reduced GMV, consistent with reports of higher incidence and burden of cardiovascular disease in men compared to women.^55^ Arterial stiffness is also independently associated with WMHV^56^ across all measures and demographic groups. These results provide important data pertaining to who is more likely to have more deleterious effects of arterial stiffness on overall brain imaging, independent from blood pressure and antihypertensive control. Given evidence showing arterial stiffness preceding the development of diseases including hypertension,^7^, ^8^ these results may represent early arterial mechanisms that may contribute to brain changes putting individuals at risk for dementia. More work is needed to better understand these possible factors.

Our findings are consistent with what has been proposed and replicated in Vascular Contributions to Cognitive Impairment and Dementia (VCID).^57^ They suggest that increased arterial stiffness directly influences brain structural damage through effects on penetrating arterioles of the brain, especially those feeding the white matter. This altered structure and function may ultimately disrupt clearance of protein and other debris from the brain.^58^ Studies have shown that vascular changes do not just act via an additive effect on AD/ADRD but play a role in the exacerbation and possibly the propagation of AD‐related pathology. As part of a positive feedback loop, aberrant vascular dynamics can occur following amyloid accumulation, thus resulting in weakened arterioles, particularly with the presence of cerebral amyloid angiopathy, poor brain perfusion, and increased small‐vessel pulsatility that further exacerbate amyloid deposition and vascular deterioration.^9^, ^13^, ^14^ Nonetheless, we were unable to interrogate the relationship between arterial stiffness and tau pathology. Prior studies using PET and cerebrospinal fluid analyses suggest that higher measures of aortic stiffness and pressure pulsatility were associated with greater rhinal and entorhinal tau burden in older adults^10^ and with higher levels of total‐ and phosphorylated tau.^59^ Both studies showed a relationship independent of Aβ burden, suggesting a potential pathway with vascular hemodynamics, warranting further exploration. Finally, a recent study showed an association between pulse wave velocity and cerebrospinal fluid biomarkers including phosphorylated tau‐181, which is sensitive to Aβ and, to a lesser extent, tau pathology.^60^

This study has limitations. Not all individuals completed both arterial stiffness measures; sensitivity analyses were completed with overlapping measures and found similar results. Global measures of brain morphology may have lower sensitivity and reduce specificity of brain‐related effects from arterial stiffness. Future work in MESA will examine cerebral blood flow and other measures of cerebral small vessel disease. Additionally, amyloid PET was only available in a subset of participants at this examination, limiting analyses of the interactions by gender and race/ethnicity for amyloid status. All analyses were cross‐sectional and therefore the temporal ordering of the development of arterial stiffness and brain MRI and amyloid PET markers could not be examined. PET and MRI scans were not acquired at the same time. We did not have tau PET to interrogate the relationship between arterial stiffness measures and tau pathology in relation to‐ and independent of amyloid, nor did we have carotid pressure to calibrate carotid stiffness measures; therefore, we relied on brachial pressure, which can overestimate central blood pressure.^61^ Not all MESA sites participated in carotid ultrasound at Exam 6, especially those enrolling Chinese American participants. Future studies focused on these specific subgroups can address these limitations.

This study demonstrated that carotid and regional arterial stiffness measures are differentially related to brain imaging abnormalities seen in AD/ADRD. Carotid ultrasound methods, including carotid distensibility and elastic modulus, were associated with WM injury (WMHV and WMFA), but not amyloid deposition. Regional stiffness measures showed mixed associations with WM injury where CAVI was associated with WMHV only in a basic model, while heart‐ankle PWV was associated with WMHV in both models and WMFA in a basic model only. CAVI was associated with amyloid deposition in the basic model and was attenuated by adjustment of cardiovascular disease, suggesting shared pathways. Arterial stiffness measures differentially captured interactions between demographic and ADRD‐related neuroimaging abnormalities. These findings support prior evidence that regional, not carotid, stiffness is more strongly linked to AD‐related changes. Future studies should directly interrogate the structural and hemodynamic mechanisms connecting stiffness with AD/ADRD biomarkers and cognition.

## CONFLICT OF INTEREST STATEMENT

The authors declare no conflicts of interest. Author disclosures are available in the supporting information

## CONSENT STATEMENT

The research protocol was approved by the local Institutional Review Board, with informed consent obtained for all participants.

## Supporting information



Supporting Information

Supporting Information
