# Supplementary material for: Carotid and regional arterial stiffness and dementia‐related imaging biomarkers in the Multi‐Ethnic Study of Atherosclerosis (MESA)
Source: Alzheimers Dement. 2025 Oct 17;21(10):e70688. doi: 10.1002/alz.70688 (PMC12531903; doi:10.1002/alz.70688)
Supplement: Supplementary file 2 — Supporting Information [file ALZ-21-e70688-s001.docx]

Supplemental Figure 1. Correlation Matrix Between Arterial Stiffness measures


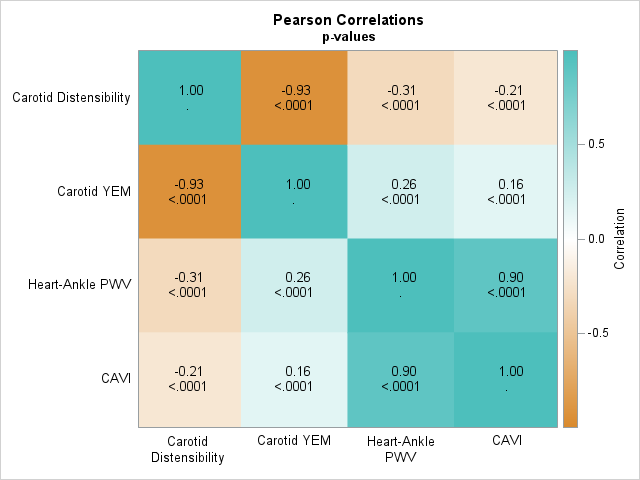


Log transformed carotid Ultrasound measures; PWV = pulse wave velocity; YEM = Youngs Elastic Modulus; CAVI = Cardio-ankle vascular index

| **Supplemental Table 1.** MESA Exam 6 demographic, vascular, cognitive, and neuroimaging measures among participants who have Neuroimaging data and arterial stiffness measures from VaSera (Regional; N=1301) or Carotid Ultrasound (Carotid; N=528), or both (N=517), and any participants excluded from analyses (N=1993). | | | | | | | | | | | | | | |
| --- | --- | --- | --- | --- | --- | --- | --- | --- | --- | --- | --- | --- | --- | --- |
|  | | | **MESA participants with Regional Stiffness and Neuroimaging** | | | **MESA participants with Carotid Stiffness and Neuroimaging** | | | **MESA participants with both Regional and Carotid Stiffness and Neuroimaging** | | | **Exam 6 participants Excluded from all Analytic Samples** | | |
|  |  |  | (n = 1301) | | | (n = 528) | | | (n = 519) | | | (n = 1993) | | |
|  | | | **Total N** | N / Mean / Median | % / SD / IQR | **Total N** | N / Mean / Median | % / SD / IQR | **Total N** | N / Mean / Median | % / SD | **Total N** | N / Mean / Median | % / SD |
| Age Exam 6 | | mean, SD | 1301 | 72 | 8 | 528 | 72 | 8 | 519 | 72 | 8 | 1993 | 76 | 9 |
| Education | ≤ High School | n, % | 1301 | 330 | 25% | 528 | 134 | 25% | 519 | 131 | 25% | 1993 | 630 | 32% |
|  | > High School |  |  | 971 | 75% |  | 394 | 75% |  | 388 | 75% |  | 1363 | 68% |
| Gender | Female | n, % | 1301 | 690 | 53% | 528 | 288 | 55% | 519 | 283 | 55% | 1993 | 1065 | 53% |
|  | Male |  |  | 611 | 47% |  | 240 | 45% |  | 236 | 45% |  | 928 | 47% |
| Race/ | White | n, % | 1301 | 547 | 42% | 528 | 229 | 43% | 519 | 225 | 43% | 1993 | 771 | 39% |
| Ethnicity | Chinese |  |  | 161 | 12% |  | 0 | 0% |  | 0 | 0% |  | 259 | 13% |
|  | Black |  |  | 342 | 26% |  | 167 | 32% |  | 163 | 31% |  | 505 | 25% |
|  | Hispanic |  |  | 251 | 19% |  | 132 | 25% |  | 131 | 25% |  | 458 | 23% |
| Family History of  Dementia | No | n, % | 1301 | 961 | 74% | 528 | 395 | 75% | 519 | 387 | 75% | 1993 | 1582 | 79% |
|  | Yes |  |  | 340 | 26% |  | 133 | 25% |  | 132 | 25% |  | 411 | 21% |
| APOE-e4 | No | n, % | 1301 | 903 | 69% | 528 | 366 | 69% | 519 | 359 | 69% | 1993 | 1398 | 70% |
|  | Yes |  |  | 339 | 26% |  | 140 | 27% |  | 138 | 27% |  | 454 | 23% |
|  | Missing |  |  | 59 | 5% |  | 22 | 4% |  | 22 | 4% |  | 141 | 7% |
| Current Smoking Status | No | n, % | 1301 | 1228 | 94% | 528 | 492 | 93% | 519 | 483 | 93% | 1993 | 1881 | 94% |
|  | Yes |  |  | 73 | 6% |  | 36 | 7% |  | 36 | 7% |  | 112 | 6% |
| Hypertension | No | n, % | 1301 | 561 | 43% | 528 | 227 | 43% | 519 | 224 | 43% | 1993 | 694 | 35% |
| Medication | Yes |  |  | 740 | 57% |  | 301 | 57% |  | 295 | 57% |  | 1299 | 65% |
| SBP | | mean, SD | 1301 | 126 | 20 | 528 | 128 | 19 | 519 | 128 | 19 | 1993 | 129 | 22 |
| Diabetes Status | Normal | n, % | 1301 | 726 | 56% | 528 | 302 | 57% | 519 | 297 | 57% | 1993 | 1006 | 50% |
|  | IFG |  |  | 304 | 23% |  | 113 | 21% |  | 113 | 22% |  | 448 | 22% |
|  | Untreated Diabetes |  |  | 53 | 4% |  | 21 | 4% |  | 21 | 4% |  | 73 | 4% |
|  | Treated  Diabetes |  |  | 218 | 17% |  | 92 | 17% |  | 88 | 17% |  | 466 | 23% |
| ***Arterial Stiffness measures*** | | | | | | | | | | | | | | |
| Carotid Distensibility (1/mmHg) | | median / IQR | 521 | 0.003 | 0.002 | 528 | 0.003 | 0.002 | 519 | 0.003 | 0.002 | 540 | 0.002 | 0.002 |
| Carotid Youngs Elastic Modulus (mmHg/mm) | | median / IQR | 521 | 1170.45 | 840.60 | 528 | 1175.55 | 848.62 | 519 | 1171.67 | 843.56 | 540 | 1332.68 | 991.60 |
| Heart-ankle PWV (m/s) | | median / IQR | 1301 | 8.40 | 1.45 | 519 | 8.47 | 1.51 | 519 | 8.47 | 1.51 | 1662 | 8.48 | 1.52 |
| Cardiac-ankle vascular index (CAVI) (units) | | median / IQR | 1301 | 8.94 | 1.66 | 519 | 9.05 | 1.65 | 519 | 9.05 | 1.65 | 1656 | 9.03 | 1.66 |
| ***MRI*** | | | | | | | | | | | | | | |
| Total GMV (μl) | | mean / SD | 1301 | 596993 | 65924 | 528 | 595498 | 67389 | 519 | 595441 | 67448 | 66 | 586905 | 71412 |
| Hippocampal volume (μl) | | mean / SD | 1301 | 3552 | 428 | 528 | 3523 | 427 | 519 | 3522 | 430 | 66 | 3496 | 478 |
| WMHV (μl) | | median / IQR | 1287 | 2805 | 6342 | 526 | 2930 | 6176 | 517 | 2927 | 6208 | 64 | 2819 | 8417 |
| WMFA | | median / IQR | 1182 | 0.39 | 0.04 | 471 | 0.40 | 0.04 | 465 | 0.40 | 0.03 | 48 | 0.39 | 0.02 |
| ***PET Imaging*** | | | | | | | | | | | | | | |
| GM Centiloids > 12.2 | No | n / % | 264 | 184 | 70% | 152 | 107 | 70% | 147 | 103 | 70% | 25 | 19 | 76% |
|  | Yes |  |  | 80 | 30% |  | 45 | 30% |  | 44 | 30% |  | 6 | 24% |
| APOE-e4 = apolipoprotein epsilon 4; PWV = pulse wave velocity; GMV = gray matter volume; WMHV= white matter hyperintensity volume; WMFA = white matter fractional anisotropy; SBP = Systolic Blood Pressure. Hippocampal volume indicates mean bilateral hippocampal volume; IFG = Impaired Fasting Glucose | | | | | | | | | | | | | | |

| **Supplemental Table 2.** Associations between Carotid and Regional Stiffness measures across demographic factors | | | | | | | | | | | | | |
| --- | --- | --- | --- | --- | --- | --- | --- | --- | --- | --- | --- | --- | --- |
|  | | **Carotid Distensibility** | | | **Carotid Youngs Elastic Modulus** | | | **Heart-ankle PWV** | | | **CAVI** | | |
|  | |  |  |  |  |  |  |  |  |  |  |  |  |
|  | | **N = 1068** | | | **N = 1068** | | | **N = 2957** | | | **N = 2957** | | |
| **Parameter** | | **Estimate** | **SE** | **p-value** | **Estimate** | **SE** | **p-value** | **Estimate** | **SE** | **p-value** | **Estimate** | **SE** | **p-value** |
| Intercept | | 4.42 | 0.30 | **< 0.001** | -3.19 | 0.32 | **< 0.001** | -4.99 | 0.17 | **< 0.001** | -4.24 | 0.18 | **< 0.001** |
| Age at Exam 6 | | -0.03 | 0.003 | **< 0.001** | 0.02 | 0.004 | **< 0.001** | 0.03 | 0.002 | **< 0.001** | 0.05 | 0.002 | **< 0.001** |
| Gender | Men | 0.03 | 0.05 | 0.572 | -0.005 | 0.06 | 0.935 | 0.49 | 0.03 | **< 0.001** | 0.37 | 0.03 | **< 0.001** |
|  | Women | Ref | | | Ref | | | Ref | | | Ref | | |
| Race/Ethnicity | Chinese | NA | | | NA | | | -0.05 | 0.06 | 0.408 | -0.03 | 0.06 | 0.637 |
|  | Black | -0.25 | 0.07 | **< 0.001** | 0.10 | 0.791 | 0.191 | -0.01 | 0.095 | 0.79 | -0.08 | 0.05 | 0.10 |
|  | Hispanic | -0.27 | 0.08 | **< 0.001** | 0.22 | 0.065 | **0.007** | -0.09 | 0.077 | 0.07 | -0.09 | 0.05 | 0.08 |
|  | White | Ref | | | Ref | | | Ref | | | Ref | | |
| *APOE*-ε4 Status | Yes | 0.03 | 0.07 | 0.627 | -0.05 | 0.07 | 0.445 | 0.01 | 0.04 | 0.770 | 0.02 | 0.04 | 0.677 |
|  | Missing | 0.06 | 0.11 | 0.562 | -0.13 | 0.12 | 0.258 | 0.02 | 0.07 | 0.731 | 0.06 | 0.07 | 0.431 |
|  | No | Ref | | | Ref | | | Ref | | | Ref | | |
| Current Smoking Status | No | Ref | | | Ref | | | Ref | | | Ref | | |
|  | Yes | 0.02 | 0.12 | 0.846 | -0.03 | 0.12 | 0.831 | 0.13 | 0.07 | **0.045** | 0.10 | 0.07 | 0.184 |
| Diabetes Status | Normal | Ref | | | Ref | | | Ref | | | Ref | | |
|  | IFG | -0.17 | 0.07 | **0.011** | 0.19 | 0.07 | **0.010** | -0.006 | 0.04 | 0.886 | -0.006 | 0.04 | 0.876 |
|  | Untreated Diabetes | -0.24 | 0.14 | 0.091 | 0.33 | 0.15 | **0.034** | 0.07 | 0.08 | 0.409 | 0.08 | 0.09 | 0.359 |
|  | Treated Diabetes | -0.21 | 0.07 | **0.005** | 0.18 | 0.08 | **0.027** | 0.16 | 0.04 | **< 0.001** | 0.15 | 0.05 | **< 0.001** |
| Hypertension Medication | No | Ref | | | Ref | | | Ref | | | Ref | | |
|  | Yes | -0.14 | 0.06 | **0.020** | 0.19 | 0.06 | **0.003** | -0.14 | 0.03 | **< 0.001** | -0.15 | 0.04 | **< 0.001** |
| Education | ≤ High School | Ref | | | Ref | | | Ref | | | Ref | | |
|  | > High School | 0.07 | 0.06 | 0.251 | -0.07 | 0.07 | 0.318 | -0.001 | 0.04 | 0.975 | 0.009 | 0.04 | 0.820 |
| SBP | | -0.01 | 0.001 | **< 0.001** | 0.01 | 0.002 | **< 0.001** | 0.02 | 0.001 | **< 0.001** | 0.006 | 0.001 | **< 0.001** |
| Models adjusted for Age at exam 6, Race/Ethnicity, Gender, *APOE*-ε4, Site, Smoking status, education, systolic blood pressure, Diabetes Status. SE= standard error. CAVI = cardiac-ankle vascular index; PWV = pulse wave velocity; *APOE*-ε4 = apolipoprotein epsilon 4; IFG = impaired fasting glucose; SBP = systolic blood pressure | | | | | | | | | | | | | |

| **Supplemental Table 3.**   Associations Between Arterial Stiffness Measures and Brain Outcomes on participants who had data available for all stiffness measures. | | | | | | | | | | | | | |
| --- | --- | --- | --- | --- | --- | --- | --- | --- | --- | --- | --- | --- | --- |
|  | | **Total GMV (mm^3^)** | | | **Hippocampal volume (mm^3^)** | | | **WMH volume*** | | | **WM Fractional Anisotropy** | | |
|  |  | (N = 519) | | | (N = 519) | | | (N=517) | | | (N=465) | | |
| **Arterial Stiffness Measure** | | **Estimate** | **SE** | **p-value** | **Estimate** | **SE** | **p-value** | **Estimate** | **SE** | **p-value** | **Estimate** | **SE** | **p-value** |
| **Carotid Measures** | | | | | | | |  | | | | | |
| Carotid  Distensibility | Model 1 | 1836 | 1470 | 0.212 | -20.43 | 15.78 | 0.196 | -0.18 | 0.06 | **0.005** | 0.003 | 0.001 | **0.029** |
|  | Model 2 | 2283 | 1543 | 0.140 | -8.37 | 16.63 | 0.615 | -0.14 | 0.07 | **0.033** | 0.002 | 0.001 | 0.112 |
| Carotid Youngs Elastic Modulus | Model 1 | -1645 | 1439 | 0.254 | 16.98 | 15.46 | 0.273 | 0.20 | 0.06 | **0.001** | -0.004 | 0.001 | **0.004** |
|  | Model 2 | -2158 | 1508 | 0.153 | 4.89 | 16.25 | 0.764 | 0.17 | 0.07 | **0.009** | -0.003 | 0.001 | **0.022** |
| **Regional Measures** (N = 519) | | | | | | | |  | | | | | |
| Heart-ankle  PWV | Model 1 | 446 | 1443 | 0.757 | 14.63 | 15.49 | 0.345 | 0.12 | 0.06 | 0.052 | -0.002 | 0.001 | 0.105 |
|  | Model 2 | 412 | 1581 | 0.795 | 5.95 | 17.00 | 0.727 | 0.06 | 0.07 | 0.418 | -0.002 | 0.001 | 0.237 |
| CAVI | Model 1 | 188 | 1420 | 0.895 | 10.40 | 15.24 | 0.495 | 0.03 | 0.06 | 0.582 | -0.0006 | 0.001 | 0.596 |
|  | Model 2 | 384 | 1441 | 0.790 | 8.25 | 15.49 | 0.595 | -0.001 | 0.06 | 0.983 | -0.0003 | 0.001 | 0.813 |
| Model 1: Age at exam 6, Race/Ethnicity, Gender, APOE4, Site, ICV (For volumes) | | | | | | | | | | | | | |
| Model 2: Age at exam 6, Race/Ethnicity, Gender, APOE4, Site, Smoking status, education, systolic blood pressure, Diabetes Status, ICV (For volumes), | | | | | | | | | | | | | |
| anti-hypertension medication; CAVI = cardiac-ankle vascular index; PWV = pulse wave velocity; YEM = Youngs Elastic Modulus; GMV = gray matter volume; WMH = white matter hyperintensity; Carotid Ultrasound full dataset; N = 1068; CAVI Measures full dataset, N = 2955; Hippocampal volume indicates mean bilateral hippocampal volume; * = ln(WMH+0.001) | | | | | | | | | | | | | |

| **Supplemental Table 4.** Associations between Arterial Stiffness Measures and Amyloid PET Positivity | | | | | |
| --- | --- | --- | --- | --- | --- |
| **Subset of Overlap with Carotid and Regional Arterial Stiffness Measure** **and PET** (N=147) | | | | | |
| **Arterial Stiffness Measure** | | **Positive/ Total** | **Odds Ratio** | **95% CI** | **p-value** |
| **Carotid Measures** | | | | | |
| Carotid Distensibility | Model 1 | 44 / 147 | 0.78 | (0.49, 1.25) | 0.304 |
|  | Model 2 | 44 / 147 | 0.76 | (0.46, 1.25) | 0.277 |
| Carotid Youngs Elastic Modulus | Model 1 | 44 / 147 | 1.28 | (0.81, 2.05) | 0.308 |
|  | Model 2 | 44 / 147 | 1.37 | (0.83, 2.26) | 0.218 |
| **Regional Measures** | | | | | |
| Heart-ankle PWV | Model 1 | 44 / 147 | 1.65 | (1.05, 2.57) | **0.029** |
|  | Model 2 | 44 / 147 | 1.80 | (1.07, 3.02) | **0.027** |
| CAVI | Model 1 | 44 / 147 | 1.67 | (1.11, 2.52) | **0.015** |
|  | Model 2 | 44 / 147 | 1.65 | (1.04, 2.64) | **0.035** |
| Model 1: Age at exam 6, Race/Ethnicity, Gender, APOE4, Site | | | | | |
| Model 2: Age at exam 6, Race/Ethnicity, Gender, APOE4, Site, Smoking status, education, systolic blood pressure, anti-hypertension medication, Diabetes status; CAVI = cardiac-ankle vascular index; PWV = pulse wave velocity; Reference Group: Centiloids < 12.2 (n=103). | | | | | |

| **Supplemental Table 5.** P-values for tests of moderation by risk factors of the association of carotid measures of arterial stiffness with neuroimaging | | | | | |  |
| --- | --- | --- | --- | --- | --- | --- |
| **Carotid Measures** (N = 528) | | **GMV** | **Hippocampal Volume** | **WMHV** | **WMFA** |  |
|  |  |  |  |  |  |  |
| Carotid Distensibility | Age | 0.85 | 0.74 | 0.88 | 0.66 |  |
|  | *APOE*-ε4 | 0.91 | 0.57 | 0.31 | 0.17 |  |
|  | Gender | 0.92 | 0.94 | 0.48 | **0.047** |  |
|  | Race | **0.02** | **0.02** | 0.80 | 0.08 |  |
|  | Family history of Dementia | 0.97 | 1.00 | 0.68 | 0.61 |  |
| Carotid Youngs Elastic Modulus | Age | 0.97 | 0.69 | 0.83 | 0.85 |  |
|  | *APOE*-ε4 | 0.45 | 0.29 | 0.42 | 0.29 |  |
|  | Gender | 0.94 | 0.56 | 0.95 | 0.15 |  |
|  | Race | **0.04** | 0.06 | 0.90 | 0.15 |  |
|  | Family history of Dementia | 0.63 | 0.88 | 0.41 | 0.62 |  |
| Models adjusted for Age at Exam 6 (split at median age of 73), years of education, Race, and Gender. Brain Volumes were also adjusted for ICV; GMV = gray matter volume; WMHV = white matter hyperintensity volume; WMFA = white matter fractional anisotropy; *APOE*-ε4 = apolipoprotein epsilon 4 computed with 3 levels (0 – No; 1 – Yes; 2 – Missing); Hippocampal volume indicates mean bilateral hippocampal volume. Models did not include adjustment for site. | | | | | |  |
|  |  |  |  |  |  |  |

| **Supplemental Table 6.** P-values for tests of moderation by risk factors of the association of regional measures of arterial stiffness with neuroimaging | | | | | |
| --- | --- | --- | --- | --- | --- |
| **Regional Measures** (N = 1301) | | **GMV** | **Hippocampal** | **WMHV** | **WMFA** |
|  |  |  | **Volume** |  |  |
| Heart-ankle PWV | Age | 0.82 | 0.57 | 0.81 | 0.93 |
|  | *APOE*-ε4 | 0.78 | 0.82 | 0.45 | 0.25 |
|  | Gender | **0.02** | 0.99 | 0.71 | 0.13 |
|  | Race | 0.49 | 0.68 | 0.85 | 0.71 |
|  | Family history of Dementia | 0.36 | 0.08 | 0.79 | 0.43 |
| CAVI | Age | 0.60 | 0.51 | 0.43 | 0.98 |
|  | *APOE*-ε4 | 0.95 | 0.79 | 0.66 | 0.47 |
|  | Gender | **0.03** | 0.49 | 0.81 | 0.08 |
|  | Race | 0.84 | 1.00 | 0.82 | 0.41 |
|  | Family history of Dementia | 0.86 | 0.18 | 0.31 | 0.27 |
| Models adjusted for Age at Exam 6 (split at median age of 73), race, *APOE*-ε4, and gender; Brain volume models also adjusted for ICV. GMV = gray matter volume; WMHV = white matter hyperintensity volume; WMFA = white matter fractional anisotropy; CAVI = cardiac-ankle vascular index; *APOE*-ε4 = apolipoprotein epsilon 4 computed with 3 levels (0 – No; 1 – Yes; 2 – Missing); Hippocampal volume indicates mean bilateral hippocampal volume. Models did not include adjustment for site. | | | | | |
